# Emergency separation of conjoined twins in a tertiary hospital in Indonesia: three case reports

**DOI:** 10.1186/s12910-023-00895-z

**Published:** 2023-02-25

**Authors:** Andi Ade Wijaya Ramlan, Raihanita Zahra, Kshetra Rinaldhy, Christopher Kapuangan, Rahendra, Komang Ayu Ferdiana, Ahmad Yani

**Affiliations:** 1 Department of Anesthesiology and Intensive Care, Faculty of Medicine, Universitas Indonesia, Jl Diponegoro No. 71, Jakarta Pusat, DKI, Jakarta, 10430 Indonesia; 2 Dr. Cipto Mangunkusumo Hospital, Jakarta, Indonesia; 3 Pediatric Surgery Division, Department of Surgery, Faculty of Medicine, Universitas Indonesia, Jakarta, Indonesia

**Keywords:** Conjoined twins, Emergency separation, Ethics, Case report

## Abstract

**Background:**

Emergency separation of conjoined twins is performed when one twin is already dead or dying and threatens the survival of the other. The particular decision to perform an emergency separation of conjoined twins provides an ethical dilemma that needs special attention. Adding to the complexity of surgical and postsurgical management in emergency separation, ethical and sociocultural aspects further complicate decision-making.

**Case presentation:**

From 1987 to 2022, 18 conjoined twin separations were performed in our centre. This paper describes three conjoined twin emergency separations. In the first case of thoracoomphalopagus babies at nine days of age, one baby was diagnosed with necrotizing enterocolitis with frequent desaturation and seizures, and the other baby was healthy. Emergency separation was performed on the twelfth day of age; unfortunately, neither baby survived the surgery. In the second case, emergency separation was performed on the 110th day of life due to sepsis in one baby. The nonseptic twin passed away six hours after surgery, while the septic twin died 12 days after surgery due to wound dehiscence and abdominal sepsis. The third case was of an omphalopagus conjoined twin with a parasitic twin. The healthy baby was deemed nonviable but found to be healthy upon birth. Immediate emergency separation was performed at 2 h of age. The living baby survived the surgery but passed away two months later.

**Conclusions:**

When separation is deemed necessary to save one twin, it becomes difficult to apply standard ethical medical reasoning. The decision to separate results in most cases in very high-risk surgeries with poor outcomes during surgery and postsurgery. Compounded by the complexity of the case, sociocultural and religious aspects further add to the dynamics of decision-making. A multidisciplinary team must work together with a health ethics committee and navigate through this ethical conundrum with the patient and family at its decision-making centre to decide on the best plan of care.

## Background

Conjoined twins are a rare congenital anomaly that presents two connected children with imprecisely understood anatomy and physiology. Conjoined twins have an incidence of 1:1000 pregnancies and higher in Africa and Asia, approximately 1:14,000 to 1:25,000. These conditions bring unique challenges to anaesthesiologists regarding separation surgery [1–3]. Since the first successful craniopagus separation in 1987, our hospital has become one of Indonesia's conjoined twin surgery centres [4]. By 2022, the institution had separated 18 conjoined twins from 45 recorded cases (Table 1).

**Table 1:** Characteristics of conjoined twins at Dr. Cipto Mangunkusumo Hospital, Jakarta, Indonesia, from 1987 to 2022

Characteristics	Number (%)	Remarks
Total	45 (100)	
Sex		
Male (%)	9 (20)	
Female (%)	36 (80)	
Type		
Omphalopagus	7 (16)	
Thoracoomphalopagus	26 (58)	
Craniopagus	4 (9)	
Ischiopagus tetrapus	4 (9)	
Ischiopagus triphus	3 (8)	
Treatment		
Nonsurgery	27 (60)	26 deaths, 1 lost to follow-up
Surgery	18 (40)	33 infants, 27 infants survived surgery
Emergency separation	5 (11)	
Planned separation	13 (29)	
Number of twins who underwent nonseparation surgery	3 (7)	2 for colostomy, 1 for insertion of tissue expander
Number of twins who underwent separation at < 28 days of age	5 (11)	6 infants survived; 2 twins underwent separation immediately after birth
Number of twins who underwent separation surgery immediately after birth	2 (4)	In one case the twin was parasitic, the other was a case of hydrops fetalis
Types of conjoined twins who underwent separation surgery		
Omphalopagus	5 (11)	8 infants, 7 infants survived
Thoraco-abdominopagus	9 (20)	17 infants, 14 infants survived
Craniopagus	2 (4)	4 infants, 2 infants survived
Pyopagus	2 (4)	4 infants, 4 infants survived

Surgical separation of conjoined twins is considered a major surgery with a high mortality rate of 50% in the neonatal period and as high as 70% in emergency cases. Therefore, the procedure demands exceptional multidisciplinary teamwork involving many specialties [1, 2, 5]. Management includes preparation, surgery, and postoperative management, which includes physical and mental rehabilitation. The anatomy of the union and cross-circulation should be defined accurately by performing a thorough investigation before surgery. Preparations should include a surgical rundown and determination of methods such as tissue expansion to achieve primary closure. However, ideal preparations might not be achieved in emergency cases, putting additional risk on the patients. Emergency separation is performed when one twin is already dead or dying and threatens the survival of the other. Another condition that may lead to emergency separation is a correctable associated congenital anomaly that would be fatal if untreated, such as bowel obstruction, volvulus, or ruptured giant omphalocele.

Beyond the complexity of surgical management, emergency separation of conjoined twins also raises dilemmatic ethical issues that require special attention. In Indonesia, as in many other Asian countries, sociocultural values and religion are fundamental considerations for the team and parent, adding to the emotional burden for decision-making. This report describes three cases of conjoined twin who underwent emergency separation in our centre after multidisciplinary and ethical consideration to save at least one out of two lives. The separation surgeries were performed in 2017 (Case 3), 2019 (Case 2), and 2020 (Case 1).

## Case presentation

### Case 1

**Fig. 1:**
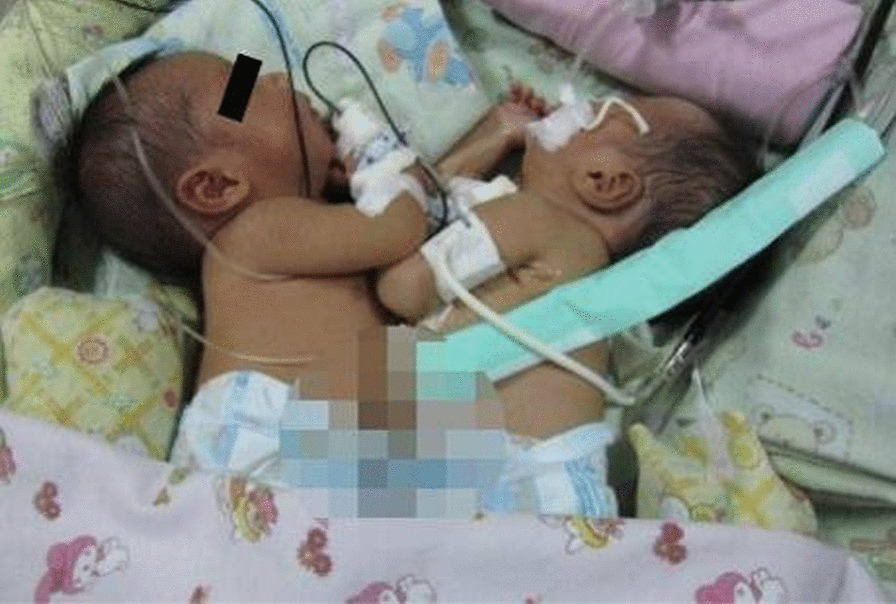
Conjoined twins in Case 1

Female thoracoomphalopagus babies (Fig. 1) were diagnosed during the third trimester of pregnancy and referred to our hospital for further treatment. The babies were delivered by emergency caesarean section at 35 weeks gestation, weighing 3400 g combined. They were joined from the thorax to the abdomen at the umbilical level. After delivery, the babies showed respiratory distress and needed mechanical ventilation in the neonatal intensive care unit (NICU). Magnetic resonance imaging (MRI) of the abdomen and thorax showed fusion of the liver without clear imaging of the gallbladders. The pericardium parietals and sternums were joined. Echocardiography showed that baby A had a normal heart, while baby B had a ventricular septal defect (VSD). No other major abnormalities were found in either baby.

After nine days of care in the NICU, baby B’s condition progressively deteriorated with frequent desaturation following seizures. She was diagnosed with necrotizing enterocolitis (NEC). However, baby A showed less progressive deterioration. On their twelfth day of life, a team meeting consisting of the multidisciplinary surgical team, neonatologists, hospital ethics committee (HEC), and hospital medical board concluded that emergency separation was considered necessary. The separation aimed to isolate each baby’s condition and save both twins. Hence, the higher expectancy for survival was for baby A. The parents and extended family of the twins were informed about the condition; therefore, after discussion with the team, the father agreed and signed the consent form.

Pre-anaesthesia preparations were undertaken by completing necessary laboratory testing and imaging. Blood products such as PRCs (packed red cells), thrombocytes, and FFP (fresh frozen plasma) were prepared before the surgery. After induction of anaesthesia, the first stage of surgery was hepatectomy, followed by thoracic resection. Hepatectomy was completed uneventfully. During thoracic resection to separate the pericardium, baby A experienced bradycardia immediately followed by ventricular fibrillation, and soon, baby B also experienced bradycardia. Resuscitation was performed, including internal cardiac compression for both babies. However, resuscitation failed to provide a response, and both babies passed away.

### Case 2

**Fig. 2:**
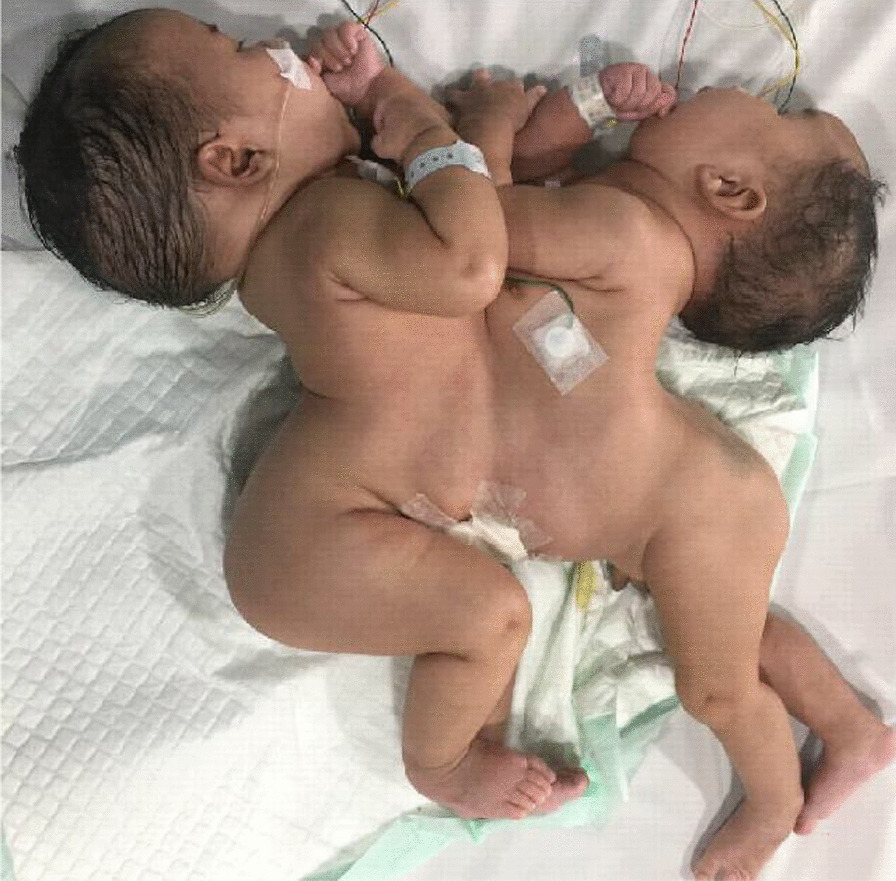
Conjoined twins in Case 2

Female thoracoomphalopagus conjoined twins, weighing 4202 g combined, were delivered at 35 weeks gestation by caesarean section. The twins were joined from the thoracic to umbilical level (Fig. 2). The APGAR scores for babies A and B were 7/9 and 3/6, respectively, with baby A being more prominent in size. The babies were admitted to the NICU and received respiratory support using mechanical ventilation. Furthermore, the babies were difficult to wean and remained on mechanical ventilation for three weeks.

The computed tomography (CT) scan showed a collapse of the left lung caused by stenosis of the left bronchus. A CT scan showed fusion of the left liver lobes with passive hepatic congestion, shunting between the distal hepatic vein in baby A and a small branch of the hepatic vein in baby B. There was fusion of the duodenum and possible intestinal fusion at the terminal ileum level. Echocardiography indicated a VSD in baby A and a VSD with a PDA (patent ductus arteriosus) in baby B. The babies also shared the parietal pericardium. No other significant abnormalities were found in either baby. On her 84th day, baby B suffered from neonatal sepsis with an uncompromised condition. However, baby A remained healthy. Multiple discussions between the multidisciplinary team member and the parents were held to decide on further treatment for the twins. The parents insisted on surgery, whereas the physician team believed that the condition of baby B could be improved through medication. A respected figure in the family further weighed in on the discussion advocating for surgery. After consideration from the multidisciplinary team, parents and the respected figure, emergency separation was deemed crucial to isolate baby A from septic baby B. Surgery was performed on their 110th day of life. The patients were considered to have ASA III-IV status. Massive bleeding occurred during liver resection. Resuscitation was performed on both babies, and high-volume transfusions were given. Both babies survived the surgery and were transferred to the NICU. Baby A passed away 6 h after the surgery due to secondary coagulopathy. Baby B survived the critical condition post-surgery. However, she passed away twelve days post-surgery because of wound dehiscence leading to abdominal sepsis.

### Case 3

**Fig. 3:**
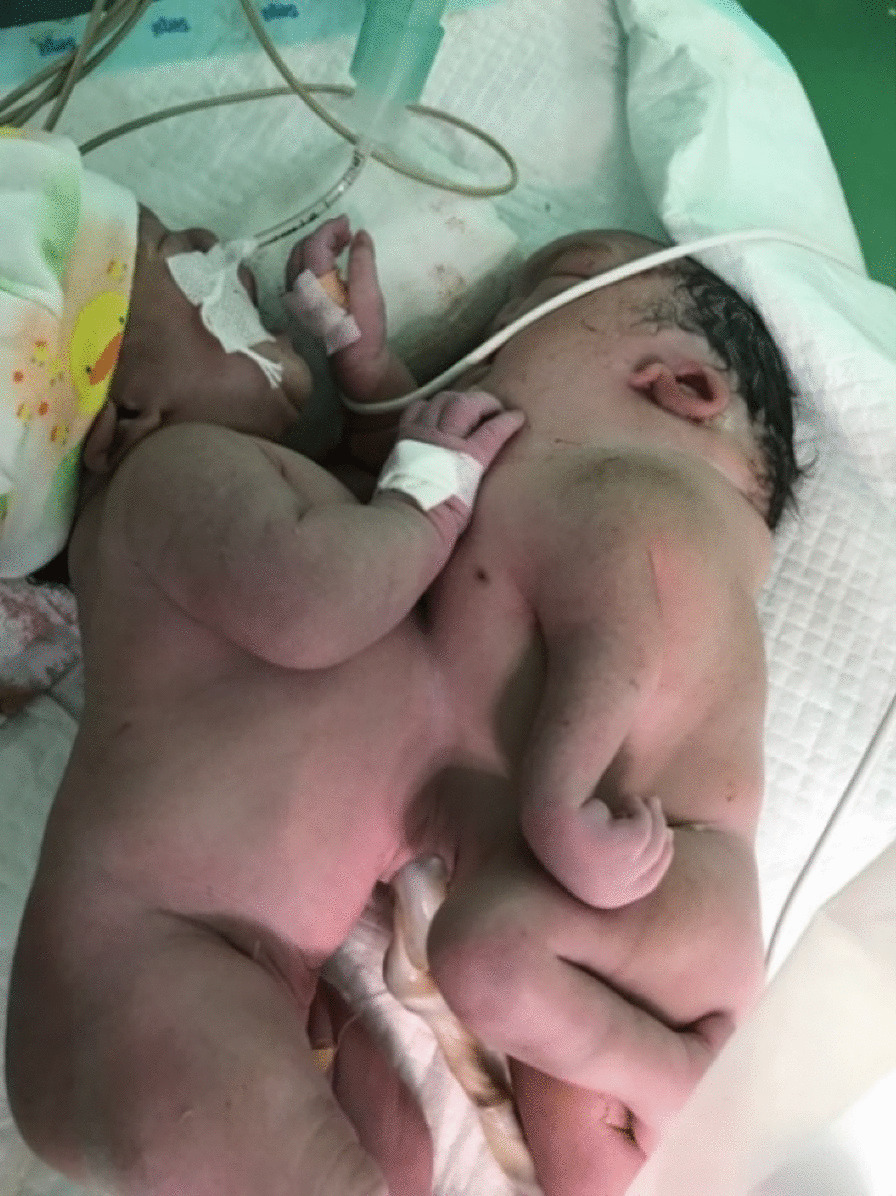
Conjoined twins in Case 3

Female omphalopagus asymmetrical parasitic conjoined twins (Fig. 3), weighing 4000 g combined, were delivered at 37 weeks gestation by caesarean section. The twins were diagnosed conjoined from the antenatal examination at 35 weeks of pregnancy. Foetal USG showed that one baby died with a pulseless heart, and the other baby had severe cardiac anomalies. The babies were joined from the abdominal part from the epigastric to the upper umbilical area. A multidisciplinary team consisting of paediatric surgeons, paediatric anaesthetists, nurses, the hospital ethics committee, and hospital medical management were gathered and expected that the twins would be nonviable.

At delivery, we found that the dead baby was parasitic, and to our surprise, we found the other baby crying and had an APGAR score of 6/8. Immediate echocardiography was performed in the operating room and showed that the living baby had a complete atrial and ventricular septal defect and patent ductus arteriosus with pulmonary atresia and had a differential diagnosis with severe pulmonary stenosis.

Consent for separation was immediately obtained from the HEC and father. Preoperative preparations were undertaken, and the baby’s condition was improved by giving dextrose intravenous fluid, cleaning, and warming the baby. Induction of anaesthesia started 2 h after birth using ketamine 1 mg/kg, fentanyl 2 mcg/kg, and atracurium 0.3 mg/kg, and a central venous catheter was inserted. Anaesthesia was maintained using sevoflurane, fentanyl 2 mcg/kg/hr, and intermittent atracurium. The deceased baby did not receive any treatment. The haemodynamic condition of the living baby was not stable because of the bleeding that occurred during liver resection. However, there was no critical event during the surgery. The patient was admitted to the NICU for further observation 2 h and 45 min after surgery. The surgery was determined to be successful. However, the baby passed away in the second month due to a worsening cardiac condition.

## Discussion and conclusions

The high mortality rate of surgical separation of conjoined twins is determined by the cause of emergency separation, time for surgery planning, and the babies’ conditions. In cases of conjoined twin, risk is increased due to the lack of preparation time for surgery and the presenting physiology of immature neonates. Therefore, separation must be carried out only if there is a risk of death to either one or both babies [1–3]. Emergency separation usually occurs when one twin dies or when a life-threatening condition presents before separation. These conditions include damage to a connecting bridge (e.g., ruptured omphalocele), deterioration of both twins because of haemodynamic and respiratory compromise (e.g., congestive heart failure, difficult-to-treat respiratory or cardiovascular involvement), and conditions where one twin threatens the other twin (e.g., intestinal obstruction, obstructive uropathy, sepsis). Previous studies reported that most conjoined twins did not survive or died soon after birth [1, 2]. Stuart argued that emergency separation is indicated even if the degree of the connection makes separation surgery dangerous [3]. Other than in emergency cases, separation surgery usually takes place after the neonatal stage [6]. In our institution, only 18 of 45 twins (40%) underwent separation surgery, with 27 out of 33 infants surviving the surgery (Table 1). Based on previous reports, the mortality rate of nonseparated twins during the neonatal period is reported to range between 60 and 100% [7, 8]. In our centre, out of 27 twins that were not separated, 26 died, while one twin was lost to follow-up. Most conjoined twins presented with multiple congenital anomalies and severe cardiac problems that led to rapid deterioration and eventual death.

Sukhla et al. reported a case of a thoracoomphalopagus conjoined twin who survived emergency separation after 1 h of surgery, a 30-ml blood transfusion and ten days of care in the NICU. Emergency separation was required after one twin died 2 h after delivery and threatened the other living twin [9]. Watanatittan et al. reported 11 cases of surgical separation, and three pairs of twins underwent emergency separation [10]. Of the three pairs that underwent emergency separation surgery, one pair underwent emergency separation after one baby died, and the other baby died one hour after surgery. For the other two pairs that underwent emergency surgery, only one infant survived the surgery. To date, our institution has performed five emergency separations. Three of them are presented in this case series. The two other emergency separations were ethically uneventful. One case was of healthy twins that were joined minimally by the liver and peritoneum. The case was not a true emergency. The separation aimed to give both twins a normal life as early as possible, considering the minimal connection. The surgery was performed a week after admission to our hospital at 28 days of age. The last case was a case of omphalopagus babies with one twin who died one hour post-delivery because of hydrops fetalis. Emergency separation was performed in the NICU to save the living baby.

In our cases, all three pairs of conjoined twins underwent emergency surgery for a life-threatening illness, either in one or both babies. In the first case, one baby was near death, while the other twin's condition was good. In the second case, the twin underwent emergency separation because one baby deteriorated due to sepsis. In the third case, the twin underwent emergency separation surgery because of intrauterine death of the other twin (parasitic conjoined twins), which compromised the surviving twin's condition. Both of these cases showed that when one baby deteriorated, the other baby was compromised and prone to rapid deterioration.

As with all ethical issues in medicine, a principle-based approach is most commonly used (beneficence, nonmaleficence, justice and autonomy). As with most conjoined twin cases, we struggled with the ethics of separation of a conjoined twin. Beneficence and nonmaleficence are the backbones of medical decisions, which makes conjoined twin separations in particular an ethical conundrum. However, assessing a case through ethical principles can lead to conflicting choices, depending on which twin’s perspective is analysed. Therefore, an approach based on ethical principles alone is not sufficient [11].

When separation is necessary for one twin to survive, it is not easy to apply standard ethical reasoning. In cases one and two, we acted on beneficence, acknowledging that emergency separation was needed to benefit the patient, particularly one twin. However, for the benefit of one twin, the decision was made with full knowledge that the separation would harm the other twin. Justice in particular should be addressed in cases where there is unequal distribution of limbs or nonvital organs. Autonomy, respecting the decision of the patients, in this case the parents, also provides another ethical dilemma [12]. In Indonesia, obtaining consent has several obstacles related to sociocultural considerations and the religious beliefs of parents. It is common in Indonesian families to have a patriarchal or matriarchal figure responsible for decision-making. The patriarchal or matriarchal figure is not restricted to the nuclear family and may often involve extended families and religious figures. While not legally constraining, the decision made by these figures weigh heavily on the decision-making of the parents. In some cases, the authoritative figure may agree with the medical team’s decision, while in some cases, they may be against the medical team’s decision. Discrepancy between the medical team, authoritative figure and parents leads to a delayed and prolonged decision-making period, possibly affecting the outcome of the twin, particularly in Case 2, in which the decision to separate was made 26 days after the deterioration of one twin. The decision to separate the twins in Case 3 was made within minutes of delivery. The decision was primarily made by the HEC with the father’s consent, as the mother was still under sedation post-caesarean section. The institution by law gives hospital management with HEC approval authority over lifesaving procedures. Although the father signed the consent form, the nature of the emergency case did not allow us to give thorough information regarding the procedure and prognosis. In this case, the principle of autonomy was not fully implemented even though we made sure to conduct a meeting after the surgery to address any issues the family had. Perhaps with hindsight, the parents should have been informed of the small possibility of a viable birth and the need for emergency separation.

As physicians, our mission is to do no harm and ensure the child’s best interests while also honouring the parents’ right to make decisions for the child. In our centre, every ethical conflict is discussed with the HEC, where the decision is based on clinical judgement and sociocultural and religious considerations. Each decision to separate is based on the complexity of the anomaly, which is also related to the ability of the surgical and postsurgical teams, considering the available hospital resources. In contrast to nonemergency separations, quality of life and long-term outcomes were not sufficiently considered. In our three cases, the decision for surgery was made after multiple discussions with the HEC, the parents, and all team members. The parents of the twins in cases 1 and 2 agreed with the decisions and signed the consent form; in Case 3, only the father signed the consent form. However, a challenge in our cases in Indonesia was the dynamics of adding another party (extended family or respected figure) to the decision-making process.

Preparation for parents is essential before any other preparation, considering that the decision they make involves the possibility that one or both infants may die during the surgery. Preoperative preparation includes patient and environment preparation. Patient preparation for emergency surgery must be performed to investigate the area of fusion and the patient's general condition prior to surgery. Preparation of the environment includes equipment, location, transportation to the surgery location, and temperature in the operating theatre. After that, the multidisciplinary team must first discuss the results of all investigations and decide the surgical procedure that will work best for the twins [1, 3]. Anaesthesiologists must treat each child as a separate individual, and the general principle of paediatric anaesthesia must be applied to every case. In case of emergencies, two anaesthesiologists must attend, and each anaesthesiologist must provide resuscitation for each infant so that each emergency is handled and resuscitation can be controlled [12–14]. Anaesthesia management for conjoined twins brings another challenge to anaesthesiologists. Anaesthesia management starts when surgery is needed, whether separation or nonseparation. From preparation to the postoperative period, all multidisciplinary team members must contribute to the case, and each specialist must precisely know the roles of all individuals on their team. Some say that the separation surgery of a pair of conjoined twins shows the best example of teamwork among any hospital staff [1, 3]. The nature of separation of conjoined twins presents an ethical dilemma in which it is hard to apply standard ethical medical reasoning. The decision to save one life in emergency separations must be made quickly while considering many aspects. Perhaps a well-defined ethical guideline regarding emergency separations could play a major role in deciding ethical dilemmas in the emergency separation of conjoined twins.

## Data Availability

The datasets used and/or analysed during the current study are available from the corresponding author upon reasonable request.

## Abbreviations

NICU: Neonatal intensive care unit
MRI: Magnetic resonance imaging
VSD: Ventricular septal defect
NEC: Necrotizing enterocolitis
HEC: Health ethics committee
PRC: Packed red cell
FFP: Fresh frozen plasma
APGAR: Appearance, pulse, grimace, activity, respiratory
CT: Computed tomography
PDA: Patent ductus arteriosus
ASA: American Society of Anesthesiologists
USG: Ultrasonography
OR: Operating room
